# Safety and efficacy of totally minimally invasive right colectomy in the obese patients: a multicenter propensity score-matched analysis

**DOI:** 10.1007/s13304-022-01298-2

**Published:** 2022-05-31

**Authors:** Michele Manigrasso, Mario Musella, Ugo Elmore, Marco Ettore Allaix, Paolo Pietro Bianchi, Alberto Biondi, Luigi Boni, Umberto Bracale, Elisa Cassinotti, Graziano Ceccarelli, Francesco Corcione, Diego Cuccurullo, Maurizio Degiuli, Nicolò De Manzini, Domenico D’Ugo, Giampaolo Formisano, Mario Morino, Silvia Palmisano, Roberto Persiani, Rossella Reddavid, Fabio Rondelli, Nunzio Velotti, Riccardo Rosati, Giovanni Domenico De Palma, Marco Milone

**Affiliations:** 1 Department of Advanced Biomedical Sciences, “Federico II” University of Naples, Via Pansini 5, Naples, Italy; 2 Department of Surgery, San Raffaele Scientific Institute, University Vita Salute, Milan, Italy; 3 Department of Surgical Sciences, University of Turin, Turin, Italy; 4 Dipartimento di Scienza della Salute, Università degli studi di Milano, ASST Santi Paolo e Carlo, Milan, Italy; 5 Università Cattolica del Sacro Cuore, Fondazione Policlinico Universitario Agostino Gemelli IRCCS, Largo F. Vito, Rome, Italy; 6 Department of Surgery, Fondazione IRCCS Cà Granda, Policlinico Hospital, University of Milan, Milan, Italy; 7 Department of Public Health, University of Naples Federico II, Via Pansini 5, 80131 Naples, Italy; 8 Department of General Surgery, “San Giovanni Battista” Hospital, USL Umbria 2, Foligno, Perugia, Italy; 9 Department of Laparoscopic and Robotic General Surgery, Monaldi Hospital, AORN dei Colli, Naples, Italy; 10 Department of Oncology, Surgical Oncology and Digestive Surgery, San Luigi University Hospital (S.L.U.H.), Orbassano, Turin, Italy; 11 Department of Medical, Surgical and Health Sciences, University of Trieste, Trieste, Italy; 12 Department of Clinical Medicine and Surgery, “Federico II” University of Naples, Via Sergio Pansini, 5, 80131 Naples, Italy

**Keywords:** Right colon, Cancer, Intracorporeal, Obese, Minimally invasive colectomy, Surgery

## Abstract

Despite the well-known benefits of the minimally invasive approach for the right colon cancer treatment, less is known about its feasibility and advantages in morbid obese patients. The aim of this study is to compare the postoperative outcomes after totally minimally invasive right colectomy between the obese and non-obese population. Data derived from a prospectively maintained multicenter colorectal database were analysed, dividing the enrolled patients into two groups: obese (BMI > 29.99) patient group and non-obese patient group. Data about gender, age, American Society of Anesthesiologists (ASA) Score, tumor characteristics, operative time, anastomosis time, extraction site, incision length, intraoperative complications, postoperative complications, postoperative recovery, specimen length and retrieved nodes were taken to assess the achievement of the oncologic standards. After a propensity score matching, a total of 184 patients was included, 92 in each group. No differences were found in terms of demographic data and tumor characteristics. Intraoperative data showed a significant difference in terms of anastomosis time in favour of non-obese group (*p *< 0.0001). No intraoperative complications were recorded and no conversion was needed in both groups. No differences were found in terms of postoperative complications. There were no differences in terms of first mobilization (*p *= 0.745), time to first flatus (*p *= 0.241) time to tolerance to liquid and solid diet (*p *= 0.241 and *p *= 0.06) and length of hospital stay (*p *= 0.817). The analysis of oncologic outcomes demonstrated adequate results in both groups. The results obtained by our study confirmed the feasibility and safety of the totally minimally invasive approach even in obese population.

## Introduction

Obesity is a global pandemic, especially in industrialized countries. Bariatric surgery is nowadays considered as the most effective approach to obtain a weight loss and a reduction in the obesity-related conditions [1–4].

Nevertheless, the increased prevalence of obesity has caused an increase even in the prevalence of colorectal cancers [5].

In the setting of right colon cancer, totally minimally invasive approach should be considered the preferred way to perform right hemicolectomy [6–8]. In this setting, several reports have demonstrated the superiority of this approach in comparison with extracorporeal anastomosis, in terms of postoperative complications and recovery outcomes [7–13]. Despite the well-known benefits of the minimally invasive approach, less is known about its feasibility and advantages in certain conditions, such as in morbid obese patients [14–16].

The aim of this study is to compare the postoperative outcomes after totally minimally invasive right colectomy between the obese and non-obese population.

## Materials and methods

After obtaining the Institutional Review Board approval of each Centre, all consecutive patients from January 2007 to December 2017 who underwent a totally minimally invasive right hemicolectomy were identified to be included in a multicenter experience. The study was conducted in compliance with the STROBE checklist [17].

Data from a prospectively maintained colorectal database, derived from high-volume colorectal centres [18], were analysed.

Each centre ensured the enrolment of at least 60 patients.

The enrolled patients were divided into two groups: obese (BMI ≥ 30 kg/m^2^) patient group and non-obese patient group.

All the included patients were operated on with a standardized totally intracorporeal right colectomy, as previously described [19, 20], and according to Enhanced Recovery After Surgery (ERAS) protocols [21, 22].

Briefly, after the division of the ileocolic pedicles and the right branches of the middle colic artery at their origin, a totally minimally invasive right hemicolectomy with an intracorporeal side-to-side ileocolic anastomosis was performed.

Demographic data (gender, age, Body Mass Index (BMI) American Society of Anesthesiologists (ASA) Score), tumor localization and TNM classification were recorded.

To assess any intraoperative challenge, data about operative time, anastomosis time, extraction site and incision length were recorded. Furthermore, intraoperative complications were recorded.

The analysed outcomes included postoperative complications, including postoperative nausea, pain, ileus, wound infection, intraluminal and extraluminal bleedings, anastomotic leakage, the need of Intensive Care Unit (ICU) and 30-day postoperative death. Furthermore, the complications were classified according their severity by the adoption of Clavien–Dindo (CD) Classification [23].

The term anastomotic leakage defined the conditions with clinical or radiologic anastomotic dehiscence, with or without the need of surgical revision. Specifically, the anastomotic leakage was classified as grade A, if resulting in no change in patients’ management; grade B, requiring active therapeutic intervention but without a surgical intervention; grade C, when a surgical re-operation was needed [24].

Any type of bleeding was considered relevant if required blood transfusions.

Other analysed outcomes were postoperative recovery, expressed as mobilization, time to first flatus and first stool, tolerance to a solid diet and length of hospital stay. Finally, data about specimen length and retrieved nodes to assess the achievement of the oncologic standards were registered.

To exclude any bias related to the allocation of each patient in the different study group, a propensity score was estimated using a multivariate logistic regression model based on age, gender, ASA Score, previous abdominal surgery, and tumour localization. One-to-one matching without replacement was performed with a 0.1 caliper width, and the resulting score-matched pairs were used in subsequent analyses.

Statistical analysis was performed using the IBM SPSS Statistics for Windows, Version 27.0 (IBM Corp, Armonk, NY). Continuous data were expressed as the means ± standard deviation (SD), and categorical variables were expressed as number and percentages. Continuous variables were compared by the Mann–Whitney *U* test and categorical variables with the Chi-square χ2 test. All results are presented as two-tailed values and a *p *< 0.05 defined a statistical significance.

## Results

The whole analysed database included 1033 patients from ten departments of surgery.

Demographic data of patients before propensity score matching are summarized in Table 1.

**Table 1:** Demographic data and tumor characteristics before propensity score matching

Characteristics	Obese (*n* = 187)	Non-obese (*n* = 846)	*p* value
Gender			**0.019**
M	113 (60.4)	429 (50.7)	
F	74 (39.6)	417 (49.3)	
Age	69.49 ± 11.55	69.21 ± 9.34	0.760
ASA Score			0.333
I	11 (5.9)	62 (7.3)	
II	102 (54.5)	495 (58.6)	
III	74 (39.6)	283 (33.5)	
IV	0 (0)	4 (0.5)	
Previous abdominal surgery	48 (27.9)	350 (41.4)	** < 0.0001**
Tumour localization			0.181
Ileo-cecal valve	39 (20.9)	176 (20.8)	
Ascending colon	102 (54.5)	515 (60.9)	
Hepatic flexure	33 (17.6)	120 (14.2)	
Proximal transverse colon	13 (7.0)	35 (4.1)	
T stage			0.771
Tx	0 (0)	3 (0.4)	
T0	2 (1.1)	13 (1.5)	
Tis	19 (10.2)	85 (10)	
T1	17 (9.1)	65 (7.7)	
T2	32 (17.1)	185 (21.9)	
T3	98 (52.4)	398 (47)	
T4a	17 (9.1)	87 (10.3)	
T4b	2 (1.1)	10 (1.2)	
N stage			**0.020**
Nx	1 (0.5)	1 (0.1)	
N0	125 (66.8)	588 (69.5)	
N1	9 (4.8)	52 (6.1)	
N1a	12 (6.4)	50 (5.9)	
N1b	18 (9.6)	64 (7.6)	
N1c	0 (0)	9 (1.1)	
N2	8 (4.3)	32 (3.8)	
N2a	4 (2.1)	38 (4.5)	
N2b	10 (5.3)	12 (1.2)	
M stage			0.543
M0	178 (95.2)	788 (93.1)	
M1	4 (2.1)	32 (3.8)	
M1a	5 (2.7)	22 (2.6)	
M1b	0 (0)	4 (0.5)	

Values are expressed as number and (percentage)

*P*-value considered significant if *p* < 0.05

*M* male, *F* Female, *ASA* American Society of Anesthesiologists, *n* number of patients in the group

**Fig. 1:**
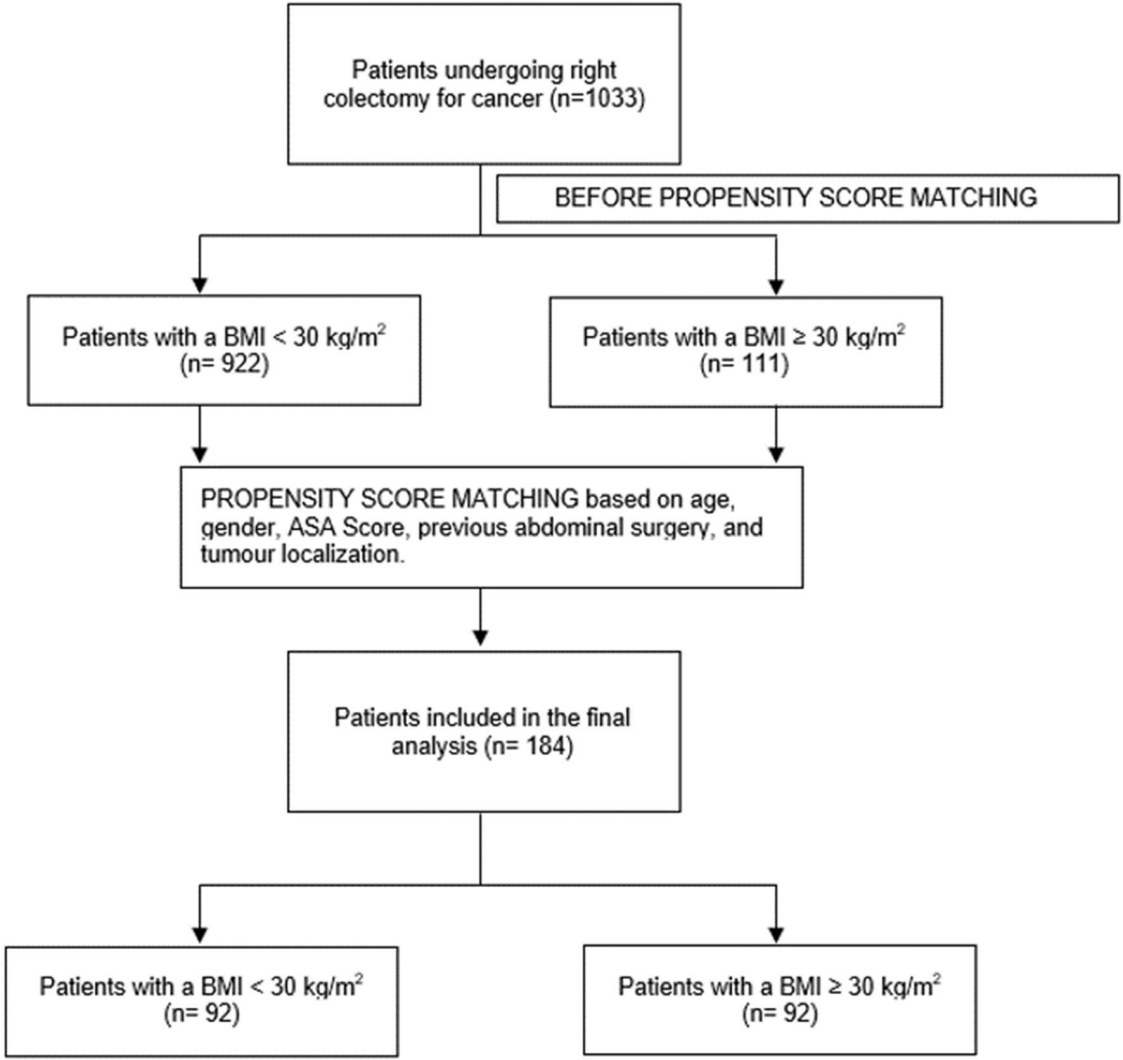
STROBE Flow Diagram of the included patients

After the propensity score matching, a total of 184 patients was included, 92 in each group. The results of STROBE Flow diagram of the inlcuded patients is represented in Fig. 1.

Mean and median BMI of the non-obese and obese patients group were 24.67 ± 2.7 and 32.31 ± 2.5, respectively, and 24.35 (18.2–29.9) and 31.3 (30–42.57) respectively, with a *p *< 0.0001. No differences were found in terms of gender (*p *= 0.615), age (obese: 69.46 ± 8.45 vs non-obese: 70.08 ± 10.43, *p *= 0.659) ASA Score (*p *= 0.580), previous abdominal surgery (*p* = 0.181), tumour localization (*p *= 0.688), T, N and M classification (*p *= 0.209, *p *= 0.110 and *p *= 0.220, respectively). Patients’ and tumours’ characteristics were summarized in Table 2.

**Table 2:** Demographic data and tumor characteristics after propensity score matching

Characteristics	Obese (*n* = 92)	Non-obese (*n* = 92)	p value
Gender			0.615
M	66 (71.7)	70 (76.1)	
F	26 (28.3)	22 (23.9)	
Age	69.46 ± 8.45	70.08 ± 10.43	0.659
BMI	32.31 ± 2.5	24.67 ± 2.7	
BMI median (range)	31.3 (30–42.57)	24.35 (18.2–29.9)	
ASA score			0.580
I	3 (3.3)	4 (4.3)	
II	52 (56.5)	45 (48.9)	
III	37 (40.2)	42 (45.7)	
IV	0 (0)	1 (1.1)	
Previous abdominal surgery	45 (48.9)	35 (38)	0.181
Tumour localization			0.688
Ileo-cecal valve	10 (10.9)	14 (15.2)	
Ascending colon	63 (68.5)	60 (65.2)	
Hepatic flexure	15 (16.3)	12 (13)	
Proximal transverse colon	4 (4.3)	6 (6.5)	
T stage			0.209
Tx	0 (0)	0 (0)	
T0	4 (4.3)	5 (5.4)	
Tis	13 (14.1)	8 (8.7)	
T1	7 (7.6)	5 (5.4)	
T2	9 (9.8)	22 (23.9)	
T3	49 (53.3)	44 (47.8)	
T4a	9 (9.8)	8 (8.7)	
T4b	1 (1.1)	0 (0)	
N stage			0.110
Nx	1 (1.1)	0 (0)	
N0	52 (56.5)	69 (75)	
N1	6 (6.5)	4 (4.3)	
N1a	8 (8.7)	3 (3.3)	
N1b	11 (12)	6 (6.5)	
N1c	0 (0)	2 (2.2)	
N2	6 (6.5)	4 (4.3)	
N2a	1 (1.1)	2 (2.2)	
N2b	7 (7.6)	2 (2.2)	
M stage			0.220
M0	83 (90.2)	87 (94.5)	
M1	7 (7.6)	2 (2.2)	
M1a	2 (2.2)	3 (3.3)	
M1b	0 (0)	0 (0)	

Values are expressed as number and (percentage). Continuous variables are expressed as mean and standard deviation. BMI has been expressed also as median and range

*M* male, *F* Female, *ASA* American Society of Anesthesiologists

Intraoperative data showed no significant differences in terms of operative time between the groups (obese 176 ± 51 min vs non-obese 180 ± 54 min, *p *= 0.622), while a significant difference was retrieved in terms of anastomosis time in favour of non-obese group (obese 19 ± 3 min vs non-obese 16 ± 4 min, *p *< 0.0001). In terms of extraction site, a significant difference was found between the two groups (*p *= 0.006), as well as the incision length (*p *< 0.0001). In details, in both group the preferred extraction site was the Pfannenstiel incision, but in the non-obese group a larger number of patients underwent a ventral midline incision (obese 13 vs non-obese 26 patients). No intraoperative complications were recorded and no conversion was needed in both groups.

Intraoperative data were summarized in Table 3.

**Table 3:** Intraoperative data

Intraoperative data	Obese (*n* = 92)	Non-obese (*n* = 92)	*p* value
Operative time	176 ± 51	180 ± 54	0.622
Anastomosis time	19 ± 3	16 ± 4	** < 0.0001**
Extraction site			**0.006**
Not specified	0 (0)	2 (1.1)	
Pfannenstiel incision	77 (83.7)	57 (61.9)	
Ventral midline incision	13 (14.1)	26 (28.2)	
Ventral out-midline incision	2 (2.2)	7 (7.6)	
Incision length			** < 0.0001**
Not specified	1 (1.1)	13 (14.1)	
< 5 cm	23 (25)	42 (45.6)	
> 5 cm but < 10 cm	68 (73.9)	35 (38)	
> 10 cm	0 (0)	2 (2.2)	
Intraoperative complications	0 (0)	0 (0)	1.000
Conversion	0 (0)	0 (0)	1.000

Continuous variables are expressed as mean ± standard deviation, values are expressed as number and (percentage)

*P*-value considered significant if *p* < 0.05

*Cm* centimetres, *n* number of patients in the group

n= number of patients in the group

The analysis of postoperative complications showed no differences between the two groups in terms of postoperative nausea (*p *= 0.305), pain (*p *= 0.246), ileus (*p *= 0.354), wound infection (*p *= 0.444), intra- and extra-luminal bleeding (*p *= 0.212 and *p *= 1.000, respectively), anastomotic leakage (*p *= 1.000), the need of ICU (*p *= 0.368) and postoperative mortality (*p *= 1.000).

In terms of treatment, one leakage required no further management, two a radiologic drainage and two a surgical re-intervention in the non-obese patients group, while two no changes in postoperative management, one a radiologic intervention and two a surgical revision in the obese patients group, respectively.

Considering the Clavien–Dindo Classification for postoperative complications, no significant differences were found in terms of CD-1 complications (obese 22 vs non-obese 20, *p *= 0.123), CD-2 complications (obese 1 vs non-obese 4, *p *= 0.368), CD-3 complications (obese 2 vs non-obese 4, *p *= 0.689), CD-4 complications (obese: 3 vs non-obese: 0, *p *= 0.311), CD-5 (obese 1 vs non-obese 1, *p *= 1.000) (Fig. 1).

The analysis of postoperative recovery outcomes showed no differences in terms of first mobilization (obese 25 ± 13 h vs non-obese 27 ± 26, *p *= 0.745), time to first flatus (obese 27 ± 26 h vs non-obese: 25 ± 12 h, *p *= 0.241) time to tolerance to liquid and solid diet (obese 39 ± 23 h vs non-obese 41 ± 25 h, *p *= 0.241 and obese 72 ± 60 h vs non-obese 89 ± 46 h, *p *= 0.06) and length of hospital stay (obese 8 ± 6 days vs non-obese 7 ± 4 days, *p *= 0.817), while a significant difference was retrieved in terms of time to first stool in favour of obese group (obese 70 ± 35 h vs non-obese 93 ± 35 h, *p *< 0.0001).

The analysis of oncologic outcomes demonstrated adequate results in both groups. In details, the length of the extracted specimen was significantly longer in the obese group (obese 29.3 ± 11.2 cm vs non-obese 24.2 ± 9 cm, *p *= 0.002), as well as the number of harvested nodes was significantly higher in the obese group (obese 24.8 ± 11.3 cm vs non-obese 19 ± 8 cm, *p *< 0.0001).

Data on postoperative complications, recovery outcomes and oncologic data are shown in Table 4.

**Table 4:** Postoperative complications, recovery outcomes and oncologic outcomes

Postoperative outcomes	Obese (*n* = 92)	Non-obese (*n* = 92)	*p* value
Postoperative complications			
Nausea	11 (12)	17 (18.5)	0.305
Pain	3 (3.3)	0 (0)	0.246
Ileus	8 (8.7)	13 (14.1)	0.354
Wound infection	5 (5.4)	2 (2.2)	0.444
Intraluminal bleeding	3 (3.3)	8 (8.7)	0.212
Extra-luminal bleeding	1 (1.1)	0 (0)	1.000
Anastomotic leakage	5 (5.4)	5 (5.4)	1.000
ICU	4 (4.3)	1 (1.1)	0.368
Death	1 (1.1)	1 (1.1)	1.000
Clavien–Dindo			
I	22 (23.9)	20 (21.7)	0.123
II	1 (1.1)	4 (4.3)	0.368
III	2 (2.2)	4 (4.3)	0.689
IV	3 (3.3)	0 (0)	0.311
V	1 (1.1)	1 (1.1)	1.000
Recovery outcomes			
Time to first mobilization (hrs)	25 ± 13	27 ± 26	0.745
Time to first flatus (hrs)	27 ± 26	25 ± 12	0.241
Time to first stool (hrs)	70 ± 35	93 ± 35	** < 0.0001**
Time to tolerance to liquid diet (hrs)	39 ± 23	41 ± 25	0.241
Time to tolerance to solid diet (hrs)	72 ± 60	89 ± 46	0.06
Length of hospital stay (days)	8 ± 6	7 ± 4	0.817
Oncologic outcomes			
Length of the extracted specimen (cm)	29.3 ± 11.2	24.2 ± 9	**0.002**
Number of harvested nodes (cm)	24.8 ± 11.3	19 ± 8	** < 0.0001**

Continuous variables are expressed as mean ± standard deviation, values are expressed as number and (percentage)

*P*-value considered significant if *p* < 0.05

*ICU* Intensive Care Unit, *hrs* hours, *cm* centimetres, *n* number of patients in the group

## Discussion

Minimally invasive right hemicolectomy can be considered nowadays as the gold standard procedure for the treatment of right colon cancer [9, 12, 13]. As known, after right colectomy the anastomosis could be performed in two different ways: in a totally intracorporeal way (totally laparoscopic intracorporeal right colectomy or laparoscopic right colectomy with intracorporeal anastomosis) or in a laparoscopic-assisted way (laparoscopic-assisted right colectomy or laparoscopic right colectomy with extracorporeal anastomosis).

Furthermore, several studies have demonstrated the superiority of the intracorporeal anastomosis over the extracorporeal reconstruction, in terms of safety and efficacy [7–13]. Similarly, several meta-analyses have shown that intracorporeal anastomosis is associated with similar postoperative outcomes over extracorporeal approach, but with significantly faster recovery, in terms of length of stay and bowel movements [6, 25–31].

However, despite these evidences, extracorporeal anastomosis continued to be performed by a large number of surgeons, probably because of the technical challenges of the intracorporeal anastomosis [32].

Furthermore, despite current data on intracorporeal anastomosis on the general population are clearly in favour of intracorporeal approach, data on the totally intracorporeal anastomosis in certain unfavourable condition, i.e. morbid obesity, are scarce and anecdotal [15, 16, 33, 34].

In 2006, Raftopoulos et al. [15] demonstrated on 45 patients, which of 13 obese, that totally laparoscopic right colectomy was safe and effective and that obesity had no effect on operative time, incision length, estimated blood loss, complications and length of hospital stay. The authors concluded that this technique had equally successful outcomes in both thin and obese patients.

In 2016, Keller et al. [16] performed a case-matched study on the adoption on single-incision laparoscopic colectomy, comparing 80 obese and 80 non-obese patients. Results showed no differences in terms of conversion rates, length of stay, complications and readmission, demonstrating that single-incision laparoscopic colectomy is safe and feasible even in the obese population.

The advantages of an intracorporeal anastomosis after right colectomy in the obese population were demonstrated by Vignali et al. [33] in their case-matched analysis on 128 patients, 64 who underwent intracorporeal anastomosis and 64 who underwent extracorporeal anastomosis. Intra-corporeal and extracorporeal anastomosis were associated with similar conversion rate, overall 30 day mortality and anastomotic leakage, while intra-corporeal anastomosis was associated with shorter recovery of bowel function, although no differences were observed in terms of length of hospital stay.

More recently, Lendzion et al. [34] proposed in 11 obese the adoption of the intra-corporeal anastomosis and the specimen extraction through natural orifice (vagina or anus). Registering only one seroma and one wound infection as postoperative complications, the authors concluded that intra-corporeal anastomosis with natural orifice specimen extraction is a good alternative in the obese patients.

According to the current literature, the intra-corporeal anastomosis seems to be feasible and safe even in the obese population. However, because of the paucity of the current data, we decided to perform this case-matched comparison to confirm the feasibility and safety of the intra-corporeal anastomosis.

In our study the obesity had no impact on the postoperative, recovery and oncologic outcomes.

First, our results confirmed the safety of the intracorporeal anastomosis in the obese population.

In fact, despite the morbid obesity could be considered a risk factor for intraoperative technical challenges, no intraoperative complications occurred in both groups, as well as the need for conversions. As expected the extraction incision was longer in the obese patients group, probably due to the rate of fatty tissue surrounding the colonic stump. Of interest, a significant difference was found between the two groups (*p *= 0.006), being a midline incision was preferred in the non-obese group. These data are in contradiction with the current literature, which demonstrated a higher rate of incisional rate in case of midline laparotomy [35]. However, this result is not dependent by any specific reason, but only by surgeons’ habits.

In terms of postoperative complications there were no differences in the two groups, i.e. postoperative nausea (*p *= 0.305), pain (*p *= 0.246), ileus (*p *= 0.354), wound infection (*p *= 0.444), intra- and extra-luminal bleeding (*p *= 0.212 and *p *= 1.000, respectively), anastomotic leakage (*p *= 1.000), the need of ICU (*p *= 0.368) and postoperative mortality (*p *= 1.000).

Even the oncologic radicality is ensured in the obese population. In fact, in both groups, the number of harvested nodes was higher than the threshold of a correct oncologic resection. Of interest, the length of colonic specimen was significantly longer in the obese group (obese 29.3 ± 11.2 cm vs non-obese 24.2 ± 9 cm, *p *= 0.002) and there is a paradoxical higher number of harvested nodes in the obese group (obese 24.8 ± 11.3 cm vs non-obese 19 ± 8 cm, *p *< 0.0001), strengthening the oncologic efficacy of a totally minimally invasive approach in these patients.

About this aspect, there are no evidences in the current literature. The longer extracted specimen, as well as the number of harvested nodes could be casual, but it could be interesting to perform further studies to confirm a correlation between obesity and colonic length or an augmented number of lymph nodes surrounding the visceral vessels.

Additionally, our results confirmed that this technique should be considered effective even in the obese patients. In fact, the comparison between the two groups demonstrated that no significance differences were found in terms of postoperative recovery outcomes, expressed as time to first mobilization (obese 25 ± 13 h vs non-obese 27 ± 26, *p *= 0.745), time to first flatus (obese 27 ± 26 h vs non-obese 25 ± 12 h, *p *= 0.241) time to tolerance to liquid and solid diet (obese 39 ± 23 h vs non-obese 41 ± 25 h, *p *= 0.241 and obese: 72 ± 60 h vs non-obese 89 ± 46 h, *p *= 0.06) and length of hospital stay (obese 8 ± 6 days vs non-obese 7 ± 4 days, *p *= 0.817).

Although the encouraging results, some limitation of this study should be addressed.

First, the retrospective design has some intrinsic inherent bias. Then, the small sample size could not ensure definitive conclusions. Finally, although several measurements have been proposed to mark technical challenges during colorectal procedures in obese patients (abdominal fat ratio, waist circumference and waist-to-hip ratio), the retrievable data obtained from our database were only on BMI. Thus, this represents an important selection bias.

Nevertheless, the results obtained by our study should be considered as a stimulus to apply a totally minimally invasive approach to right hemicolectomy even in the obese population.

However, data in the current literature remain scarce. For this reason, further high quality studies should be proposed to confirm these favourable outcomes.

## Data Availability

data are available to the corresponding Author.
